# Body Weight-Supported Treadmill Training Ameliorates Motoneuronal Hyperexcitability by Increasing GAD-65/67 and KCC2 Expression via TrkB Signaling in Rats with Incomplete Spinal Cord Injury

**DOI:** 10.1007/s11064-022-03561-9

**Published:** 2022-03-23

**Authors:** Xiangzhe Li, Xinjian Song, Lu Fang, Jie Ding, Longju Qi, Qinghua Wang, Chuanming Dong, Sheng Wang, Jiahuan Wu, Tong Wang, Qinfeng Wu

**Affiliations:** 1 Rehabilitation Medical Center, Suzhou Science & Technology Town Hospital, Gusu School, Nanjing Medical University, No.1 Lijiang Road, Suzhou, 215153 Jiangsu People’s Republic of China; 2 Department of Rehabilitation Medicine, The First Affiliated Hospital of Nanjing Medical University, No.300 Guangzhou Road, Nanjing, 210029 Jiangsu People’s Republic of China; 3 Department of Rehabilitation Medicine, Affiliated Nantong Rehabilitation Hospital of Nantong University, Nantong, 226002 Jiangsu People’s Republic of China; 4 Department of Rehabilitation Medicine, The Second People’s Hospital of Nantong, Nantong, 226002 Jiangsu People’s Republic of China; 5 Departments of Respiratory Care, Sir Run Run Shaw Hospital, College of Medicine, Zhejiang University, Hangzhou, 310016 Zhejiang People’s Republic of China; 6 Department of Orthopaedics, Affiliated Nantong Hospital 3 of Nantong University, Nantong, 226001 Jiangsu People’s Republic of China; 7 Experimental Animal Center, Nantong University, No.19 Qixiu Road, Nantong, 226001 Jiangsu People’s Republic of China; 8 Department of Anatomy, Medical College of Nantong University, No.19 Qixiu Road, Nantong, 226001 Jiangsu People’s Republic of China

**Keywords:** Spinal cord injury, Body-weight supported treadmill training, Spasticity, Tropomyosin-related kinase B, Glutamic acid decarboxylase 65/67, Potassium chloride co-transporter 2

## Abstract

Spasticity is a typical consequence after spinal cord injury (SCI). The critical reasons are reducing the synthesis of Gamma-Aminobutyric Acid (GABA), glycine and potassium chloride co-transporter 2 (KCC2) inside the distal spinal cord. The current work aimed to test whether exercise training could increase the expression of glutamic acid decarboxylase 65/67 (GAD-65/67, the key enzymes in GABA synthesis) and KCC2 in the distal spinal cord via tropomyosin-related kinase B (TrkB) signaling. The experimental rats were randomly assigned to the following five groups: Sham, SCI/phosphate-buffered saline (PBS), SCI-treadmill training (TT)/PBS, SCI/TrkB-IgG, and SCI-TT/TrkB-IgG. After that, the model of T10 contusion SCI was used, then TrkB-IgG was used to prevent TrkB activity at 7 days post-SCI. Body weight-supported treadmill training started on the 8^th^ day post-SCI for four weeks. The Hmax/Mmax ratio and the rate-dependent depression of H-reflex were used to assess the excitability of spinal motoneuronal networks. Western blotting and Immunohistochemistry techniques were utilized for measuring the expression of GAD-65, GAD-67, and KCC2. The findings revealed that exercise training could reduce motoneuronal excitability and boost GAD-65, GAD-67, and KCC2 production in the distal region of the spinal cord after SCI. The effects of exercise training were decreased after the TrkB signaling was inhibited. The present exploration demonstrated that exercise training increases GAD-65, GAD-67, and KCC2 expression in the spinal cord via TrkB signaling and that this method could also improve rats with motoneuronal hyperexcitability and spasticity induced by incomplete SCI.

## Introduction

Spasticity is one of the most frequent complications after spinal cord injury (SCI). Studies have shown [1–3] that more than 70% of patients develop various degrees of spasticity below the level of injury within 2–6 months after SCI. Mild spasticity can benefit the functional activities of patients with SCI. However, severe spasticity can lead to adverse complications such as pain, fatigue, joint contractures, pressure ulcers, and infections, any of which can seriously affect both the rehabilitation efficacy and the patient’s quality of life [4–7].

When the upper motor neurons are damaged, spasticity may occur, which is characterized by the intermittent or chronic voluntary activation of muscles [6, 8]. After SCI, the emergence of spasticity may be directly associated with an imbalance in the excitability of the spinal network [2, 9]. The strong correlation among these findings could be explained by reduced production of gamma-aminobutyric acid (GABA), glycine, and potassium chloride co-transporter 2 (KCC2), as well as reduced GABA and glycine receptors on the plasma membrane of motoneuron distal to the injured spinal cord [9–12].

Currently, baclofen is the most frequently employed first-line medication (including oral and intrathecal sustained pumping) among the common methods to clinically treat spasticity after SCI [7, 13]. Baclofen can activate GABA_B_ receptors to exert presynaptic inhibition, thereby inhibiting abnormal neuron firing and alleviating spasticity [14]. However, the beneficial effects of baclofen could be compromised due to its major adverse effects (e.g., drowsiness, fatigue, etc.) [14, 15]. More rarely, abnormal muscle tone may also occur after taking baclofen [16]. Therefore, non-pharmacological treatments (including exercise therapy) before pharmacological intervention are recommended as the first step in spasticity management [7, 15].

Both clinical and animal studies have found [10, 17–20] that activity-based functional training can improve spasticity after SCI, and this effect may be associated with the increased synthesis of the tropomyosin-related kinase B (TrkB), brain-derived neurotrophic factor (BDNF), GABA, and KCC2 in the spinal cord. Overexpression of BDNF in the damaged spinal cord increases the expression of glutamic acid decarboxylase 65/67 (GAD-65/67, the key enzymes in GABA synthesis), enhances inhibitory effects within the spinal cord, and promotes functional recovery in SCI rats [21]. When the BDNF/TrkB signaling is blocked, it can reduce the production of KCC2 within the lumbar region of the spinal cord after SCI, and weaken the alleviating effect of exercise training on spasticity in SCI rats [18, 20].

The cyclic AMP response element-binding protein (CREB) and its phosphorylated counterpart (p-CREB) may involve in increasing GAD-65/67 expressions through regulating gene transcription downstream of TrkB signaling [10]. However, further investigations are required to determine whether exercise training can modulate the expression of GAD-65/67 and KCC2 in the distal of the spinal cord, enhance spinal inhibition, and improve spasticity via TrkB signaling after SCI.

The purpose of this study was to observe whether exercise training could enhance the inhibitory effect of the damaged spinal cord through TrkB signaling and improve the spasticity of SCI rats. Thus, this study was designed to investigate the impact of exercise training on the production of GAD-65/67 and KCC2 in the distal spinal cord and to measure the excitability of intersegmental neural networks in SCI rats with or without blocking TrkB signaling.

## Materials and Methods

### Animals and Groups

Forty female adult Sprague–Dawley rats, around 210–250 g in weight (obtained from the Laboratory Animal Center of Nantong University, Nantong, Jiangsu, China), were randomly divided into five groups: sham operation group (sham group), SCI/phosphate-buffered saline (SCI/PBS) group, SCI-treadmill training/PBS (SCI-TT/PBS) group, SCI/TrkB-IgG group and SCI-TT/TrkB-IgG group. The rats were housed at a constant temperature (22 ± 2 °C), with access to food, and on a 12/12 h dark/light cycle, with 50–60% humidity. The current work was evaluated and authorized by the Medical Ethics Committee of Nanjing Medical University, and the implementation complies with the Chinese Laboratory Animal Guidelines.

### Intrathecal Catheter and Spinal Cord Injury

All rats underwent L3-4 intrathecal catheter one week before SCI [22, 23]. All rats were anesthetized with an intraperitoneal dose of 0.3 ml/100 g body-weight of 10% chloral hydrate. The L3-4 spinous procedure was identified and subjected, and a 19 g piercing needle was used to break through the subarachnoid and dura cavity. A PE-10 catheter, 6 cm long (Smiths Medical International Ltd., UK), was placed approximately 2 cm towards the head along L3-4 into the subarachnoid area. The catheter was then washed with 20 µl of sterile PBS using a 25 µl microinjector, and the PE-10 catheter's exterior port was clamped. Following surgery, all rats were returned to their former habitat and placed in a single cage.

Allen's technique was utilized for T10 incomplete SCI seven days after the intrathecal catheter procedure [23, 24]. Briefly, all rats were anesthetized as described above, then the T10 spinous process was discovered and exposed, the T10 spinous process was removed with a lamina rongeur, and the spinal cord was exposed. A device rod (10 g, 2.5 mm diameter) struck the exposed T10 spinal cord at a vertical distance of 25 mm to create the incomplete SCI model. In the sham group, the exposed spinal cord was not compressed by the rod. After surgery, all animals were given 2 ml of normal saline intraperitoneally, and bladder massage assisted voiding twice a day for 5–7 days until spontaneous voiding was established.

### Intrathecal Administration

Catheter connection and placement were accomplished similar to earlier studies [22, 23]. The recombinant human TrkB-Fc Chimera (TrkB-IgG, R&D systems, Minneapolis, USA) was used to inhibit the TrkB signaling. All rats were anesthetized again after seven days of SCI, as explained previously. The Alzet osmotic pumps were perfused with PBS formulated TrkB-IgG solution (0.25 g/l) or PBS simply. The Sham, SCI/PBS and SCI-TT/PBS groups were implanted by employing PBS perfused Alzet osmotic pumps. The SCI-TT/TrkB-IgG and SCI/TrkB-IgG groups were implanted with TrkB-IgG solution perfused pumps. The osmotic pump was connected to the PE-10 catheter by a 1 cm PE-50 catheter, and the osmotic pumps were then inserted subcutaneously in the back. The operation mentioned earlier was repeated after two weeks due to the two-week operational lifespan of the model 2002 osmotic pump. TrkB signaling was inhibited for a total of four weeks.

### Training for Treadmill

Body weight-supported treadmill training (BWSTT) was undertaken from the 8^th^ day after SCI [22, 23, 25]. Treadmill training was performed in both groups, SCI-TT/TrkB-IgG and SCI-TT/PBS groups. The bladder was manually massaged before each treadmill training session to empty it. The treadmill was set to 6 m/min for 20 min per session, twice a day, five days a week, for 4 weeks. The body weight support range was adjusted between 20 and 40%, depending on the rats’ hindlimb locomotion functional condition at training.

### H-Reflex

According to previous studies [18, 26], the rate-dependent depression (RDD) of H-reflex was performed to evaluate the spinal motoneuronal excitability and synaptic transmission at the end of the experiment. The assessment method is briefly described as follows: rats to be assessed were anesthetized as above, and the evaluation equipment was a Keypoint electromyography/evoked potential apparatus (Dantec, Denmark). For EMG recordings, bipolar wire electrodes (Cooner Wire) were placed into the interosseous muscles, with the ground electrode put into the back skin. An isolated pulse stimulator (A-M Systems) elicited H-reflexes by delivering single bipolar pulses (0.1 ms) to the tibial nerve. The current intensity was gradually increased from 0.0 mA to 0.1–1.0 mA until the M-wave amplitude remained reasonably constant and the H-wave declined.

The intensity that induced the maximum H-reflex amplitude was then used to determine the H-reflex and M-wave features and trigger RDD. Three series of 20 consecutive stimulations at 0.3, 5, and 10 Hz calculated RDD. A 5–10 min delay was included between the two tests to allow the nerve to recover to its baseline electrical state [18, 26, 27].

### Immunohistochemistry

Four rats from each group were anesthetized as described above, and the target spinal cord segments (L2-L5) were extracted and fixed with 4% paraformaldehyde. A paraffin microtome was used to make serial horizontal transverse Sects (5 µm) of spinal cord segments after being postfixed, dehydrated, and paraffin-embedded. One section was taken from every five sections and three ones were taken for each sample. Following dewaxing and hydration, H_2_O_2_ incubation, microwave repair, and blocking the serum, mouse anti-GAD 67 monoclonal antibody (MAB5406, division 1:1000, Millipore, Germany), rabbit anti-GAD 65 polyclonal antibody (ab203063, division 1:200, Abcam, USA), rabbit anti-CREB (phospho s133) monoclonal antibody (ab32096, division 1:200, Abcam, USA), rabbit anti-CREB monoclonal antibody (ab32515, division 1:500, Abcam, USA), or rabbit anti-KCC2 polyclonal antibody (ab49917, division 1:1500, Abcam, USA) was added to incubate with the sections overnight at 4 °C. The addition of rabbit anti-mouse or goat anti-rabbit antibody labelled with biotin was fulfilled the next day after PBS washing and then set to incubate at 37 °C in an incubator for 1 h. Finally, for visualization, 3,3′-diaminobenzidine-4 HCl/H_2_O_2_ (DAB, Vector Laboratories, Burlingame, CA, USA) was used.

**Fig. 1:**
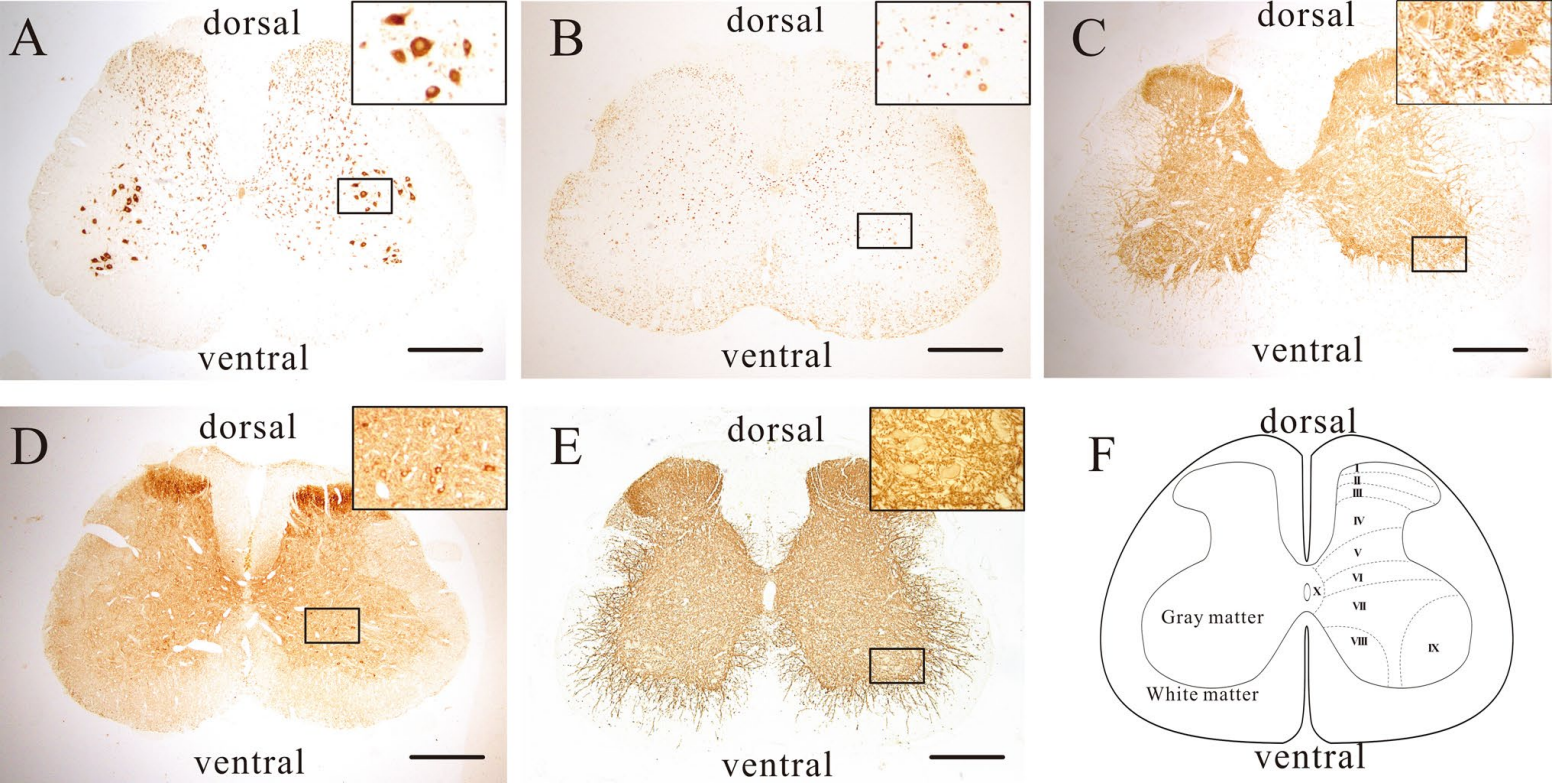
Immunohistochemical staining of CREB, p-CREB, GAD-65, GAD-67, and KCC2 in rat lumbar spinal cord horizontal transverse sections. The immunohistochemical staining reveals that **A** CREB is predominantly expressed in the neuronal cytoplasm. **B** p-CREB is upregulated in the neuron nucleus. **C** GAD-65 is widely expressed in gray matter and is associated with immunoreactive punctate structures [29, 30]. **D** GAD-67 immunopositive cells are primarily rooted in the gray matter, including the neurons in the lamina VII [29, 30], and **E** KCC2 is predominantly expressed on the cell membrane, dendritic spines and dendritic trunks of neurons in lumbar the spinal cord. **F** A schematic representation of the spinal cord's gray matter layer at the lumbar spinal cord. Scale bar: 400 μm

According to the gray matter layers of the spinal cord [28], we selected lamina VII and IX as the region of interest, and the details were as follows: lamina VII was chosen for photographing CREB/p-CREB and GAD-67, lamina IX was selected from for photographing CREB/p-CREB, GAD-65, and KCC2 (see Fig. 1).

The index of the mean density was used to determine the immunoreactivity of CREB in the neuronal cytoplasm, p-CREB in the neuron nucleus, GAD-65 puncta around the neurons, and KCC2 on the plasma membrane. Cell counting was done to determine the number of immune-positive cells for GAD-67. Densitometric analysis or cell counting was performed using Image-Pro Plus after photographing.

### Western Blot

Four rats from each group were anesthetized as described above, and the L2-L5 spinal cord segments were removed. The lumbar segments were used to prepare protein samples. The samples were put onto SDS gels containing 12% Tris/tricine. After electrophoresis, the samples were transferred to PVDF membranes. The membranes were incubated with primary antibodies: mouse anti-GAD67 monoclonal antibody (MAB5406, dilution 1:5000, Millipore, Germany), rabbit anti-GAD65 polyclonal antibody (ab203063, dilution 1:1000, Abcam, USA), rabbit anti-CREB (phospho S133) monoclonal antibody (ab32096, dilution 1:500, Abcam, USA), rabbit anti-CREB monoclonal antibody (ab32515, dilution 1:1000, Abcam, USA), rabbit anti-TrkB polyclonal antibody (dilution 1:1000, Abcam, USA), rabbit anti-BDNF monoclonal antibody (dilution 1:1000, Abcam, USA), rabbit anti-GAPDH monoclonal antibody (ab181602, 1:5000, Abcam, USA), or rabbit anti-KCC2 polyclonal antibody (ab49917, division 1:2000, Abcam) over night at 4 ℃. The ECL plus detection method was utilized to detect the immunoreactive bands after incubation with rabbit anti-mouse or goat anti-rabbit secondary antibody (1:2000) at ambient temperature for 1 h the next day. The images were then processed using Image J software.

### Statistical Analysis

For statistical analysis, IBM SPSS (v.20.0) program was utilized. One-way analysis of variance (ANOVA) was employed to examine the Hmax/Mmax ratio, immunohistochemistry, and western blot data. The Tukey HSD post hoc analysis was done if the one-way ANOVA was statistically dissimilar. For the RDD of the H-reflex, a general linear model was built, and the data were subjected to repeated measurement ANOVA. Tukey's post hoc analysis followed for comparing the values of the five groups of rats at the identical stimulation frequency. The P < 0.05 was considered statistically significant.

## Results

### Effects of BWSTT on BDNF and TrkB Expression

**Fig. 2:**
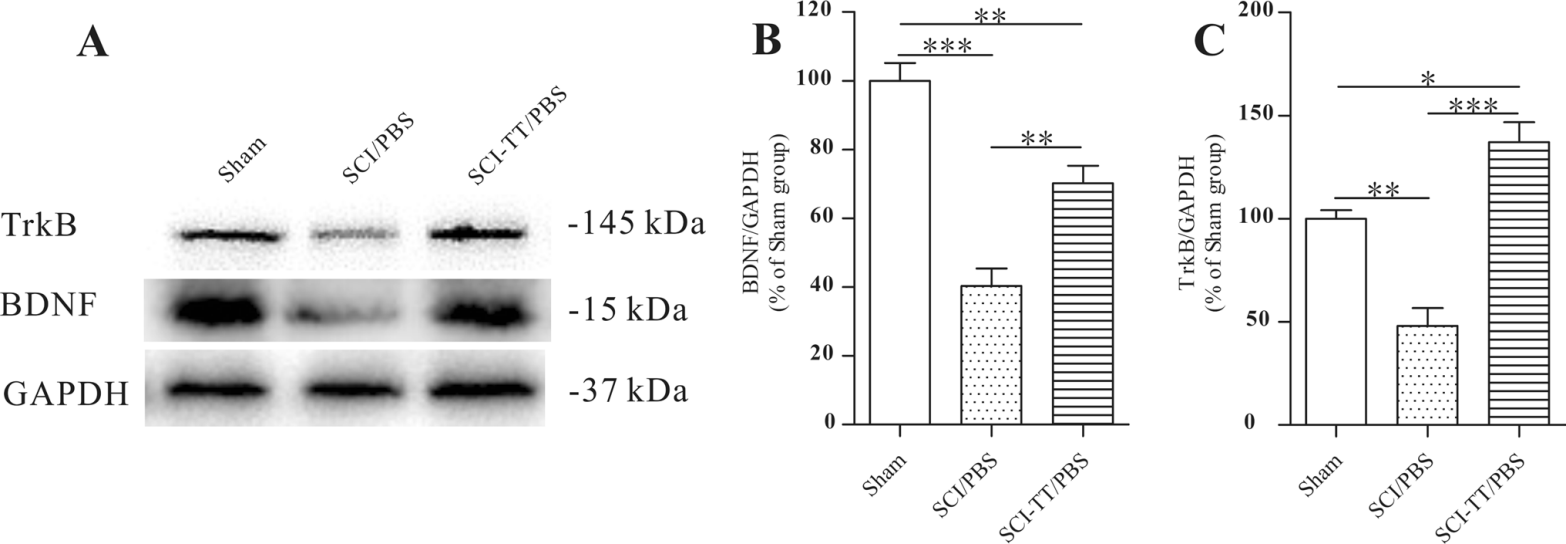
The western blot examination of BDNF and TrkB in the lumbar spinal cord. **A** Western blot analysis of BDNF and TrkB expression in the lumbar spinal cord. **B** the relative density of BDNF as a statistical graph. **C** the relative density of TrkB as a statistical graph. **P < 0.01, ***P < 0.001

The expressions of TrkB and BDNF in the injured distal spinal cord of the rats in the Sham, SCI-TT/PBS, and SCI/PBS groups were detected using Western Blot after the experiment (Fig. 2).

The results indicated that BDNF protein levels were significantly lower in the SCI-TT/PBS and SCI/PBS groups compared to the Sham group (P < 0.05). The expression of BDNF in the SCI-TT/PBS group was significantly higher than the SCI/PBS group (P < 0.05). The TrkB expression level was greater in the group of SCI-TT/PBS in comparison to the SCI/PBS and Sham groups (P < 0.05), whereas the TrkB level in the Sham group was higher when compared against the SCI/PBS group (P < 0.05).

### BWSTT Improves the Ratio of Hmax/Mmax and the RDD of the H-reflex via the TrkB Signaling

**Fig. 3:**
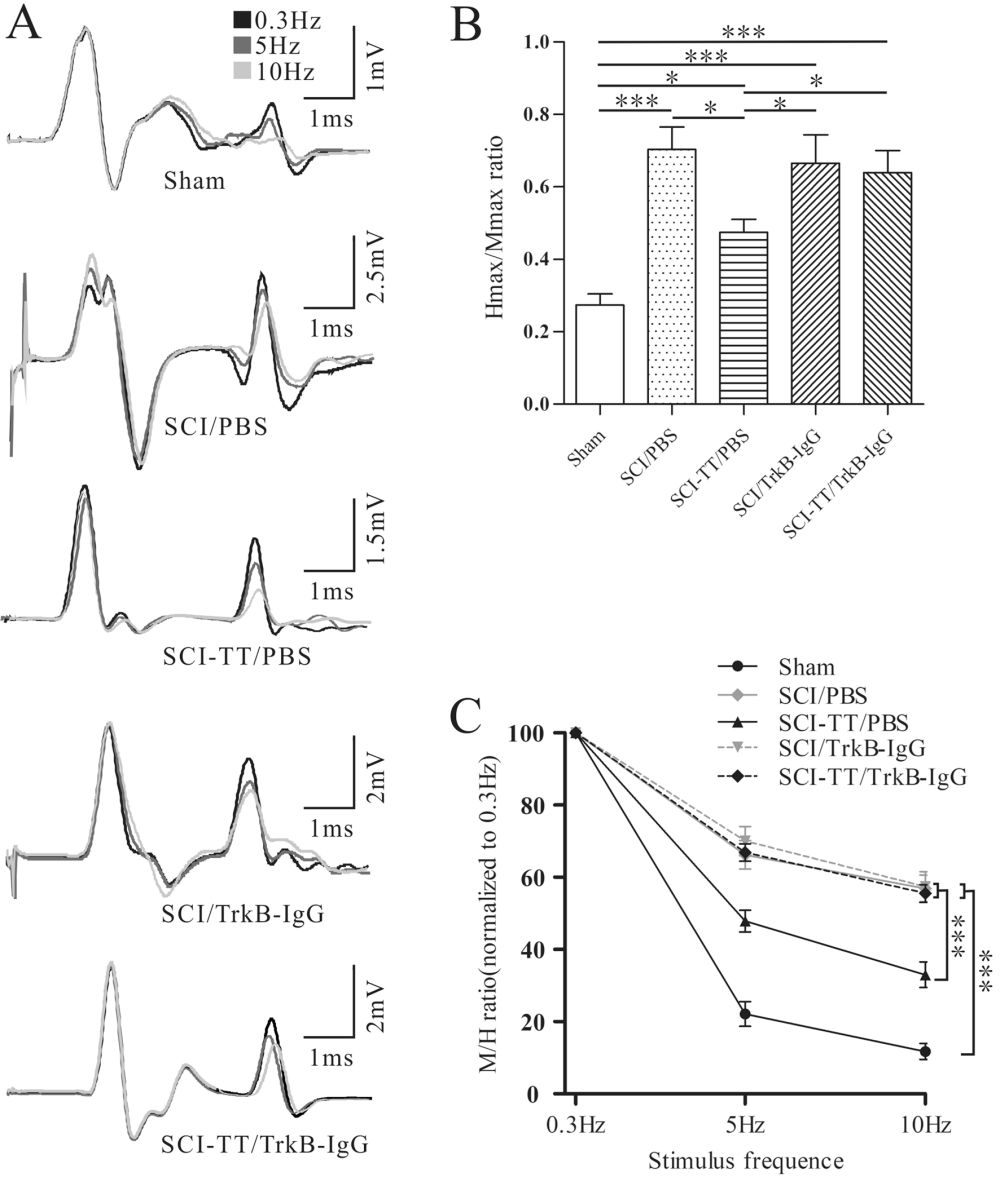
The H-reflex in five groups. **A** Typical H-reflex waveforms at 0.3, 5, and 10 Hz. **B** the Hmax/Mmax ratio graph. **C** the graph of the H-reflex RDD at various stimulus frequencies. *P < 0.05, **P < 0.01, ***P < 0.001

The ratio of Hmax/Mmax and the RDD of the hind limb H-reflex were measured at the end of the experiment to determine whether the signaling of TrkB play a role in spasticity suppression in rats with SCI after BWSTT (Fig. 3).

The Hmax/Mmax ratios of the SCI/PBS, SCI-TT/PBS, SCI-TT/TrkB-IgG, and SCI/TrkB-IgG groups were higher (P < 0.05) than that in the group of Sham. SCI-TT/PBS had a lower Hmax/Mmax ratio than the SCI/PBS, SCI-TT/TrkB-IgG, and SCI/TrkB-IgG groups (P < 0.05).

A general linear model repeated measurement ANOVA was established for analyzing the RDD of H-reflex. The results showed that the RDD of all five groups were different in stimulation frequency and group effect (P < 0.001), and there was an interaction between stimulation frequency effect and group effect (P < 0.001). At 5 Hz and 10 Hz, the H-reflex amplitude in the SCI-TT/PBS group was notably lower compared with the SCI/PBS, SCI/TrkB-IgG, and SCI-TT/TrkB-IgG groups (P < 0.05). At both 5 Hz and 10 Hz, the H-reflex amplitude of the SCI/PBS, SCI-TT/PBS, SCI/TrkB-IgG, and SCI-TT/TrkB-IgG groups was higher than that in the Sham group (P < 0.05).

### Effects of Inhibiting TrkB Signaling on BWSTT-Induced CREB and p-CREB Expression

**Fig. 4:**
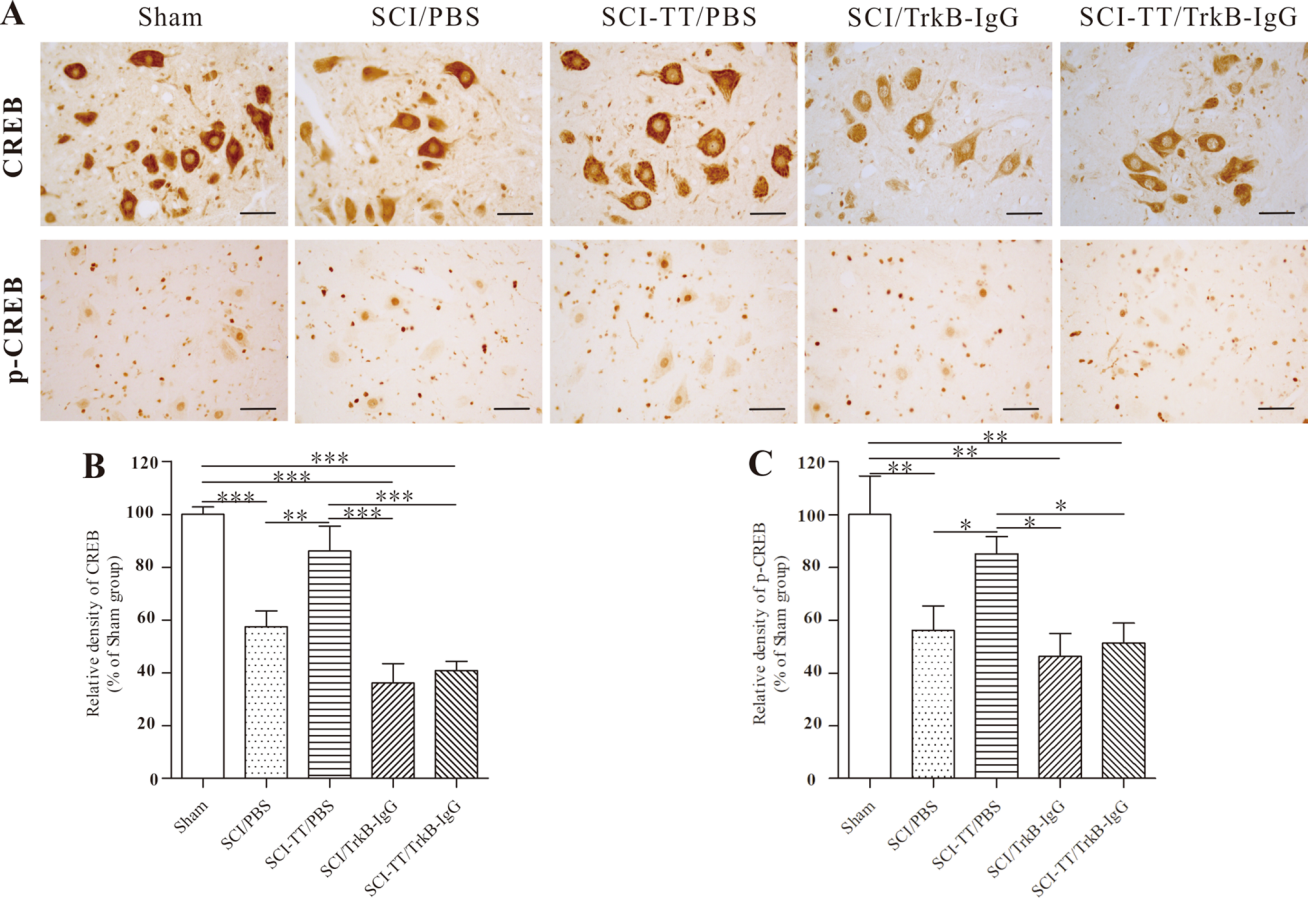
The immunohistochemical investigation of CREB and p-CREB in the distal spinal cord lamina VII. **A** Immunohistochemical analysis reveals that CREB is primarily expressed in the cytoplasm of neurons, whereas p-CREB is predominantly expressed in the nucleus of neurons. **B** and **C** the statistical graphs of the CREB and p-CREB relative densities. **P < 0.01, **P < 0.01, ***P < 0.001. Scale bar: 50 μm

**Fig. 5:**
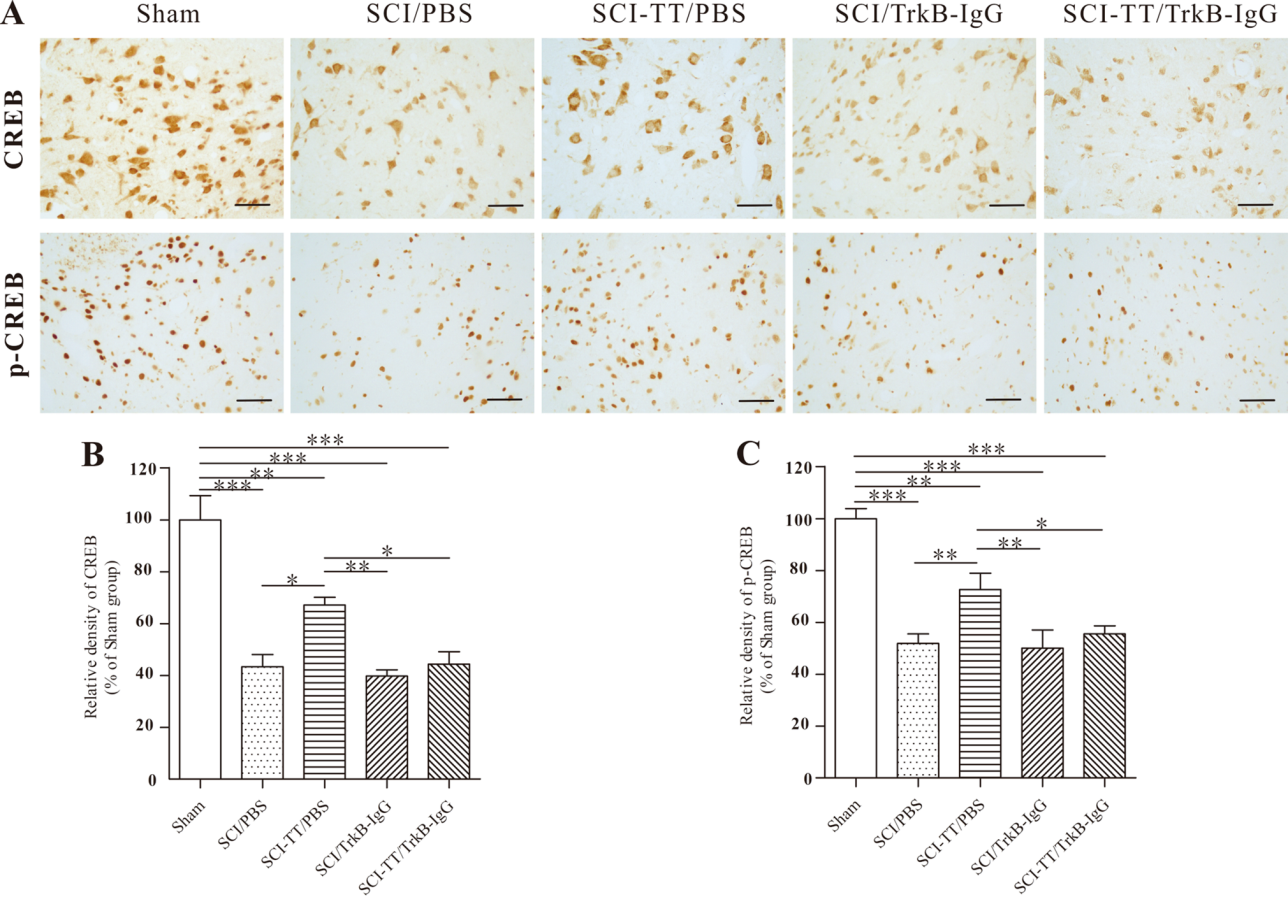
The immunohistochemical assessment of CREB and p-CREB in the distal spinal cord lamina IX. **A** Immunohistochemical analysis reveals that CREB is largely expressed in the cytoplasm of neurons, whereas p-CREB is predominantly expressed in the nucleus of neurons. **B** and **C** the statistical graphs of the CREB and p-CREB relative densities. **P < 0.01, **P < 0.01, ***P < 0.001. Scale bar: 50 μm

**Fig. 6:**
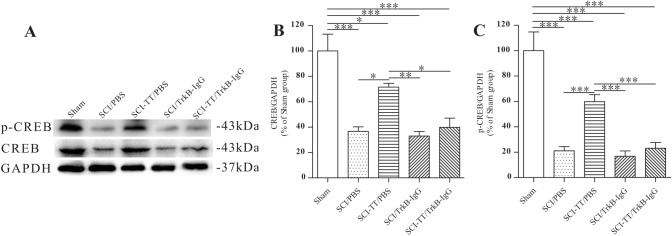
The western blot examination of CREB and p-CREB in the lumbar spinal cord. **A** Western blot picture of the CREB and p-CREB transcription factors. **B** A graph illustrating the relative density of CREB. **C** Statistical graph of p-relative CREB's density. *P < 0.05, **P < 0.01, ***P < 0.001

We observed whether blocking of TrkB signaling affects BWSTT-induced CREB and p-CREB expression in the injured distal spinal cord by western blot and immunohistochemistry analysis (Figs. 4, 5, 6).

Immunohistochemistry study of p-CREB and CREB in lamina IX revealed that the expression levels of the two proteins were lower in the SCI/PBS, SCI/TrkB-IgG, and SCI-TT/TrkB-IgG groups than in the Sham group (P < 0.05). While the expression levels of the two proteins in the SCI-TT/PBS group were significantly higher than in the SCI/PBS, SCI/TrkB-IgG, and SCI-TT/TrkB-IgG groups (P < 0.05), there were no significant differences between the SCI-TT/PBS and Sham groups (P > 0.05).

The expression levels of the two proteins were lower in the SCI/PBS, SCI-TT/PBS, SCI/TrkB-IgG, and SCI-TT/TrkB-IgG groups than in the Sham group (P < 0.05), according to immunohistochemistry of p-CREB and CREB in lamina VII and western blot of CREB and p-CREB in the distal region of the spinal cord. Furthermore, the SCI-TT/PBS group had significantly higher levels of the two proteins than the SCI/PBS, SCI/TrkB-IgG, and SCI-TT/TrkB-IgG groups (P < 0.05).

### Effects of Inhibiting TrkB Signaling on BWSTT-Induced GAD-65 and GAD-67 Expression

**Fig. 7:**
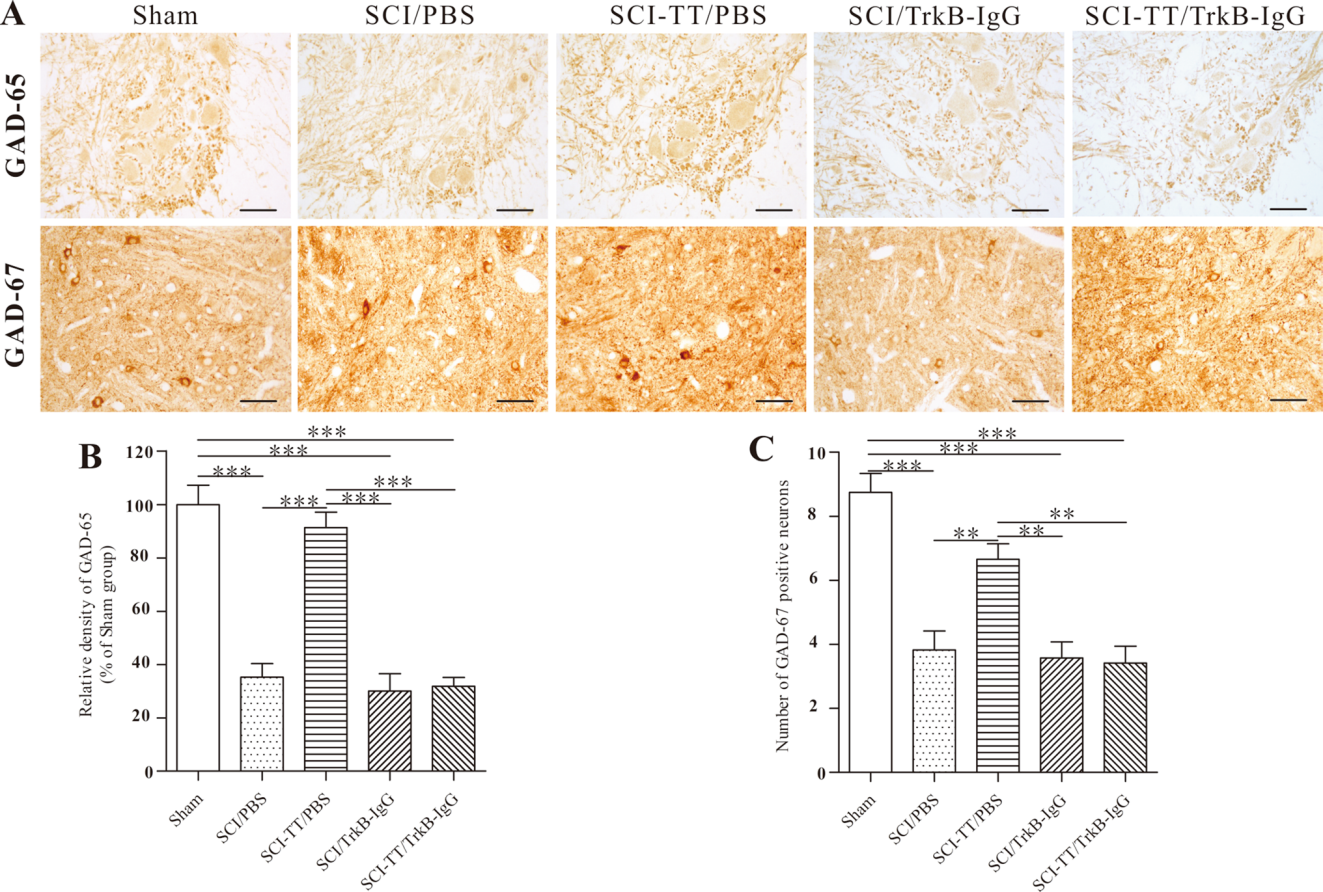
The immunohistochemical assessment of GAD-65 and GAD-67 in the lumbar spinal cord. **A** Immunohistochemical staining of GAD-65 in the lumbar spinal cord lamina IX and GAD-67 in the lumbar spinal cord lamina VII. **B** the statistical graph of the GAD-65's relative density. **C** a statistical graph of the GAD-67 immunopositive cells. **P < 0.01, ***P < 0.001. Scale bar: 50 μm

**Fig. 8:**
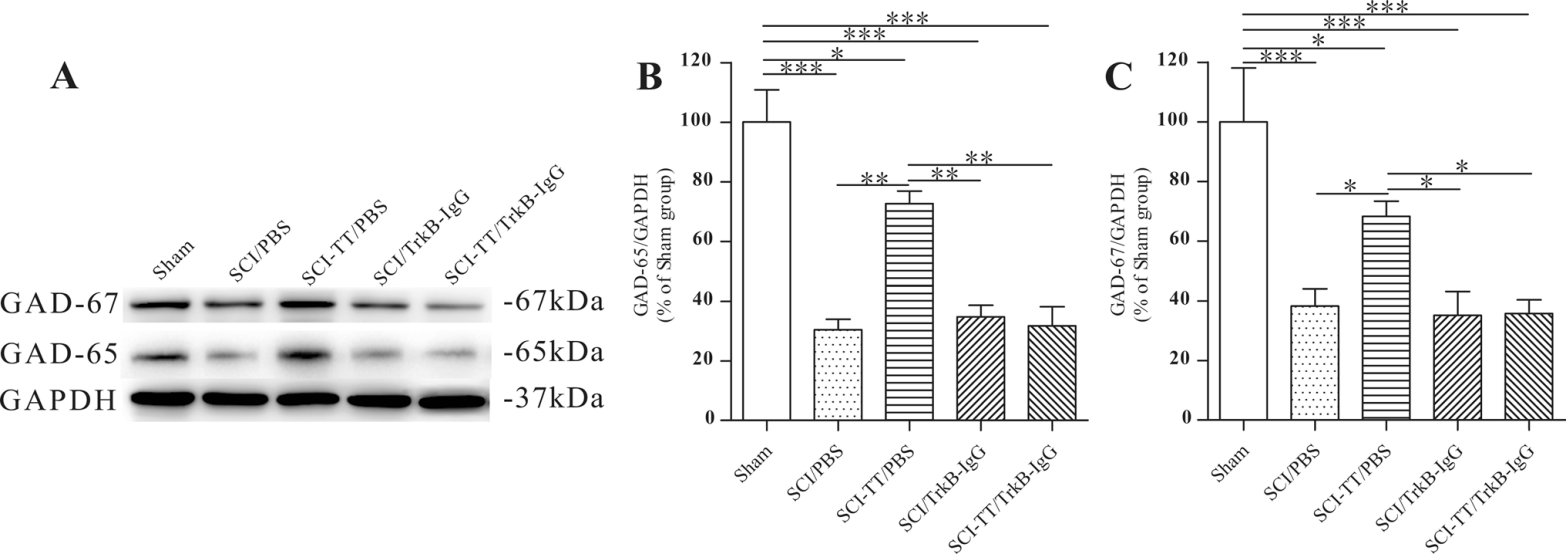
The western blot examination of GAD-65 and GAD-67 in the lumbar spinal cord. **A** GAD65 and GAD-67 western blot picture. **B** The relative density of GAD-65 is plotted statistically. **C** A statistical plot of GAD-67's relative density. *P < 0.05, **P < 0.01, ***P < 0.001

The Western blot and immunohistochemistry analyses were conducted to observe whether blocking TrkB signaling affects BWSTT-induced GAD-67 and GAD-65 expression in the injured distal spinal cord (Figs. 7, 8).

Immunohistochemistry of GAD-65 in lamina IX and GAD-67 in lamina VII revealed that the SCI/PBS, SCI/TrkB-IgG, and SCI-TT/TrkB-IgG groups had lower expression levels of the two proteins than the Sham group (P < 0.05). While the levels of the two proteins in the SCI-TT/PBS group were significantly higher than those in the SCI/PBS, SCI/TrkB-IgG, and SCI-TT/TrkB-IgG groups (P < 0.05), there were no significant differences between the SCI-TT/PBS group and the Sham group (P > 0.05).

The expression levels of the two proteins were significantly lower in the SCI/PBS, SCI-TT/PBS, SCI/TrkB-IgG, and SCI-TT/TrkB-IgG groups than in the Sham group (P < 0.05). In comparison to the SCI-TT/TrkB IgG, SCI/TrkB-IgG, and SCI/PBS groups, the levels of the two proteins in the SCI-TT/PBS group were significantly greater (P < 0.05).

### Effects of Inhibiting TrkB Signaling on BWSTT-Induced KCC2 Expression

**Fig. 9:**
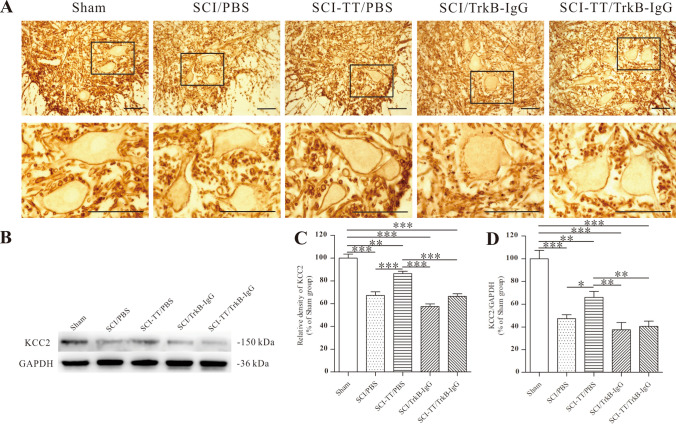
Immunohistochemistry and western blot examination of KCC2 in the lumbar spinal cord. **A** KCC2 immunohistochemistry staining in lamina IX. **B** Western blot analysis of the KCC2 protein. **C** a statistical graph depicts the relative density of KCC2 on the motor neuron membrane. **D** The relative density of KCC2 in the distal spinal cord as a statistical graph. **P < 0.01, ***P < 0.001. Scale bar: 50 μm

Both immunohistochemical and Western blot analyses were used to determine whether inhibiting TrkB signaling impacts BWSTT-induced KCC2 expression in the damaged distal spinal cord (Fig. 9).

The expression of KCC2 in the SCI/PBS, SCI-TT/PBS, SCI/TrkB-IgG, and SCI-TT/TrkB-IgG groups was substantially lower than that of the group of Sham (P < 0.05). In comparison, the expression of this protein was significantly higher in the SCI-TT/PBS group as a detailed comparison was made to the SCI/PBS, SCI/TrkB-IgG, and SCI-TT/TrkB-IgG groups (P < 0.05).

## Discussion

The current work evaluated the influence of BWSTT on spasticity in rats experiencing SCI. Our results indicated that: 1) hind limbs spasticity in rats, an increase in the Hmax/Mmax ratio, and a reduction in the RDD value could be caused by SCI; 2) Four weeks exercise training could potentially increase the level of expression for BDNF, TrkB, CREB, p-CREB, GAD-56, GAD-67, and KCC2, and reduce the Hmax/Mmax ratio and the RDD value of rats with SCI; 3) after the blocking of TrkB signaling, when compared to the SCI-TT/PBS group, the expression levels of CREB, p-CREB, GAD-56, GAD-67, and KCC2 in SCI-TT/TrkB-IgG group underwent a significant reduction, and the ratio of Hmax/Mmax and the RDD value of the H-reflex was increased.

Previous studies have shown that [10, 31, 32], after SCI, the response of motoneurons to the synaptic excitation may be amplified and prolonged, and the inhibitory mechanisms in spinal neuronal circuitry may be altered. H-reflex is widely employed to assess synaptic transmission and motoneuronal excitability from Ia afferents to motoneurons, and the Hmax/Mmax and the RDD values of the H-reflex were increased significantly after SCI [33–35]. Increased motoneuronal excitability and lower presynaptic inhibition of Ia afferents after SCI may be linked to an increase in the ratio of Hmax/Mmax and the RDD value of the H-reflex [12, 26, 35].

The GABA synthesizing enzyme (GAD-65 and GAD-67), glycine receptor α3 and KCC2 expression in the distal spinal cord is considerably reduced after SCI, contributing to presynaptic disinhibition and neuron hyperexcitability [9, 12, 34]. According to earlier studies [36–38], the excitotoxic effects of SCI (mitochondrial dysfunction and structural abnormalities produced by Ca2 + influx) might cause the degeneration of neurons in the injured distal spinal cord, including spinal anterior horn motor and GABAergic neurons.

In the central nervous system (CNS), GABA activates the ion-type GABA_A_ receptor (GABA-gated chloride channel protein that causes Cl^−^ influx and generates hyperpolarized membrane potential) and the metabotropic GABA_B_ receptor (G-Protein-coupled receptor protein that opens K^+^ channels or closes voltage-gated Ca^2+^ channels after activation, decreasing transmitter release from terminals) [39, 40]. Reduced GABA production in the CNS weakens presynaptic inhibition, resulting in aberrant excitation in the CNS, leading to spasms, neuropathic pain, epilepsy, and other problems [9, 40, 41].

The KCC2 is a co-transporter particular to neurons. According the prior research [42], the primary function of this protein in healthy adult motoneurons is the transfer of Cl^−^ to the extracellular space while also maintaining low Cl^−^ concentrations within neuronal cells. During the binding of inhibitory neurotransmitters (such as GABA and glycine) to their associated receptor channel proteins, extracellular Cl^−^ is drawn into the cell, resulting in the generation of hyperpolarization potentials and the inhibition of neuronal activity in the cell [42]. In pathological conditions, such as SCI, the distribution of KCC2 in the cell membrane of motor neurons will decrease, leading to intracellular Cl^−^ accumulation, contributing to spasticity after SCI [12, 26]. Gackière et al. [43] revealed that the level of KCC2 function in newborn mice controls the strength of postsynaptic inhibition, meaning that KCC2 downregulation after SCI contributes to an increase in flexor–extensor co-contractions. After chronic SCI, increasing KCC2 activity may help to reduce hyperreflexia and spasticity [34].

Exercise training, as a functional rehabilitation training program after SCI, has the potential to cause increased activation of sensory-motor neural circuits within the spinal cord, thereby improving neural function recovery and neural plasticity by increasing the expressions of TrkB and BDNF in the injured spinal cord [10, 18, 20, 23, 42]. It can also improve spasticity and neuropathic pain after SCI by causing an increment in the generation of GAD-65, GAD-67, and KCC2 in the spinal cord [18–20, 22, 34]. This could be due to the exercise-induced rise in BDNF/TrkB and the beneficial effect of nerve system functional remodeling [18, 22, 42].

BDNF is a neurotrophic protein that has an indispensable role in cell survival, neuronal circuit control, and neural plasticity in the CNS [44–47]. The establishment and modulation of GABAergic inhibitory neuronal circuits in the CNS of rodents may be aided by BDNF-TrkB signaling [22, 48–50]. Using the signaling of Ras/ERK (one of the TrkB signaling cascades), BDNF/TrkB can potentially stimulate p-CREB activation of the GAD-65 promoter (5.5-kb 5' GAD65-luc construct) and regulatory zones in cortical inhibitory interneurons, and the level of GAD-67 might be mediated through p-CREB within olfactory bulb cells [48, 51]. Furthermore, via modulating the PLC-1 and Shc cascade after SCI, BDNF-TrkB signaling can upregulate KCC2 expression, reducing spasticity and neuropathic pain [20, 42]. Nevertheless, the mechanistic pathway by which p-CREB promotes GAD-67 and KCC2 production needs further investigation.

In conclusion, the current study demonstrates that the expression of GAD-65/67 and KCC2 in the distal spinal cord is decreased, resulting in hyperexcitability in rats with T10 incomplete SCI. By activating the signaling of TrkB in the distal region of the spinal cord after SCI, exercise training can promote an increment in GAD-65/67 and KCC2 production in the distal area of the spinal cord and lessen the spasticity of SCI rats. However, because the current study did not examine the actual downstream signaling of TrkB signaling or evaluate the effects of other neurotrophic agents and neurotransmitters on spasticity in SCI rats, it cannot be deemed definitive. As a result, future research should focus on the regulatory effects of TrkB signaling downstream pathways on GAD-65/67 and KCC2 and the potential role of different neurotrophic factors and neurotransmitters in spasticity amelioration following SCI.

## Data Availability

Datasets analyzed during the current study are available from the corresponding author on reasonable request.
